# Lifestyle, Psychosocial and Demographic Correlates of Objective Cognitive Performance in Middle-Aged and Older Adults with Subjective Cognitive Complaints: A Cross-Sectional Baseline Analysis of the N-HANCE Cohort

**DOI:** 10.3390/brainsci16101033

**Published:** 2026-09-27

**Authors:** Diana Chira, Olivia Verișezan-Roșu, Ștefan Strilciuc, Nicoleta Jemna, Ana-Maria Buruiană, Emanuel Ștefănescu, Răzvan Marian Melinte, Sorin Claudiu Man, Dafin Fior Mureșanu

**Affiliations:** 1 Department of Neurosciences, Iuliu Hațieganu University of Medicine and Pharmacy, Victor Babeș 43, 400012 Cluj-Napoca, Romania; diana.chira@brainscience.ro (D.C.); nicoleta.jemna@brainscience.ro (N.J.); dafinm@ssnn.ro (D.F.M.); 2 RoNeuro Institute for Neurological Research and Diagnostic, Mircea Eliade 37, 400354 Cluj-Napoca, Romania; olivia.rosu@brainscience.ro (O.V.-R.); anamaria.buruiana@brainscience.ro (A.-M.B.); emanuel.stefanescu@brainscience.ro (E.Ș.); 3 Research Center for Functional Genomics, Biomedicine and Translational Medicine, Iuliu Hatieganu University of Medicine and Pharmacy, Gheorghe Marinescu 38, 400337 Cluj-Napoca, Romania; 4 Department of Translational Neurosciences, MEDFUTURE, Iuliu Hațieganu University of Medicine and Pharmacy, Gheorghe Marinescu 38, 400337 Cluj-Napoca, Romania; 5 Department of Orthopedics and Traumatology, Iuliu Hațieganu University of Medicine and Pharmacy, Victor Babeș 43, 400012 Cluj-Napoca, Romania; rmmelinte@yahoo.de; 6 Orthopedics and Traumatology Clinic “Alexandru Radulescu”, Emergency County Hospital, Traian Mosoiu 47-49, 400394 Cluj-Napoca, Romania; 7 Third Pediatric Discipline, Mother and Child Department, Faculty of Medicine, Iuliu Hatieganu University of Medicine and Pharmacy, Crișan 3-5, 400347 Cluj-Napoca, Romania; claudiu.man@umfcluj.ro

**Keywords:** cognitive performance, aging, lifestyle, subjective cognitive complaints, attention, memory

## Abstract

**Background:** Subjective cognitive complaints (SCCs) in middle-aged and older adults are recognized as early warning signs of future cognitive decline. While biological risk factors have been studied extensively, less is known about how modifiable lifestyle factors, stress, mood and sleep relate to actual performance on cognitive tests. **Objective:** To examine associations between lifestyle (physical activity, alcohol consumption), psychosocial factors (perceived stress, mood, sleep) and standardized cognitive performance in adults with SCCs. **Methods:** We conducted a cross-sectional analysis using baseline data from 276 adults aged 50–75 years enrolled in the N-HANCE study. Participants completed the Test of Attentional Performance (TAP) and Digit Span (Forward and Backward). Lifestyle and psychosocial variables were assessed using structured questions and validated self-report scales. Associations were examined using Spearman correlations and Mann–Whitney U tests, with Benjamini–Hochberg correction applied across the full family of 132 predictor–outcome comparisons, followed by linear regression models adjusted for age, sex and educational attainment. **Results:** Educational attainment showed the strongest and most consistent associations, relating to better performance across attention and memory measures assessed (ρ = 0.21 to 0.44, all q < 0.01). Older age was associated with poorer memory span and less consistent alertness. A history of depression was associated with reduced alertness and this association was essentially unchanged after adjustment (β = −0.19, *p* < 0.001). Higher BMI was associated with poorer memory span (ρ = −0.18; adjusted β = −0.16, *p* = 0.005) and higher perceived stress with poorer working memory (Digit Span Backward ρ = −0.21; adjusted β = −0.11, *p* = 0.046; *p* = 0.062 under robust standard errors). Associations involving mood and sleep quality attenuated to non-significance after adjustment, while physical activity retained only a marginal association with divided attention. Nineteen of 132 associations survived correction for multiple comparisons. **Conclusions:** In adults with SCCs, educational attainment and age, a proxy of cognitive reserve and an index of aging respectively, rather than modifiable targets, accounted for the strongest associations with objective cognitive performance. Among potentially modifiable factors, a history of depression and higher BMI showed the most robust independent associations, while associations with mood and sleep did not persist after adjustment and physical activity retained only a marginal association. These cross-sectional findings identify candidate risk and resilience markers that require confirmation in longitudinal follow-up.

## 1. Introduction

As people grow older, many begin to notice subtle changes in memory, attention or mental sharpness. These experiences, often described as subjective cognitive complaints (SCCs), are common in middle-aged and older adults. Even when formal testing shows no clear impairment, SCCs are increasingly recognized as meaningful warning signs, pointing to a heightened risk of future cognitive decline and, in some cases, even dementia [1,2]. Large European and international studies show that roughly one in four older adults without measurable cognitive impairment report noticing changes in their memory or thinking. For instance, the COSMIC consortium estimated the prevalence of subjective cognitive decline (SCD) at about 24–26% when using standardized criteria [3]. Age-related decline in several cognitive domains, including processing speed, reasoning and memory, appears to begin relatively early in adulthood rather than only in later life, with cross-sectional estimates suggesting accelerating decline after the age of 60 [4] although this pattern is far from uniform and depends on a mix of biological and lifestyle influences. Because of this, adults with SCCs represent an important group for research focused on early detection and prevention. Longitudinal evidence indicates that such complaints carry prognostic weight: individuals reporting subjective cognitive decline show an elevated risk of subsequent objective cognitive impairment and dementia and in some cases these complaints appear to precede measurable changes on formal testing [5].

Research on cognitive decline has traditionally emphasized fixed or biological risk factors such as age, genetics, vascular disease or neurodegeneration. The presence of the APOE-ε4 allele, for instance, is a well-established genetic risk factor for Alzheimer’s disease [6,7] and cerebrovascular pathology is strongly associated with poorer cognitive outcomes [8,9]. While these factors are undoubtedly important, they leave little room for intervention. On the other hand, lifestyle and psychosocial factors, such as how active people are, how much they drink, how they sleep and how they cope with stress or mood difficulties are potentially modifiable. These aspects of daily life not only affect general health, but growing evidence suggests they also play a critical role in shaping cognitive performance and brain resilience with age [10]. In interpreting such associations it is useful to distinguish several related concepts. Cognitive decline refers to the observed deterioration in performance over time, while cognitive reserve denotes the adaptability of cognitive processes that helps explain differential susceptibility to brain pathology. Brain maintenance refers to the relative absence of age-related brain change and resilience describes the capacity to sustain function despite such change [11,12]. These distinctions help explain why individuals of similar age who report comparable complaints can nevertheless differ in measured performance. Educational attainment is among the most widely used proxies of cognitive reserve, alongside occupational complexity, cognitive leisure activity and premorbid intellectual ability, and in individuals with subjective cognitive decline higher education has been associated with a lower likelihood of conversion to objective cognitive impairment [13]. It nevertheless remains an indicator of reserve rather than a direct measure of it.

Research shows, for example, that regular physical activity is a key modifiable factor for protecting cognitive health in later life. Recent findings suggest that different exercise types may benefit different domains: moderate-intensity aerobic activity appears especially supportive of executive function, while progressive resistance training has been linked to improvements in memory and attention [14,15]. On the opposite end of the spectrum, chronic stress has been repeatedly identified as a risk factor for poorer cognitive outcomes. Sustained psychological stress is linked to faster decline and a greater likelihood of developing mild cognitive impairment (MCI) or even dementia [16]. Older adults who report high levels of perceived stress tend to perform worse on memory and attention tasks, showing deterioration across multiple cognitive domains over time [17,18]. Sleep is another lifestyle factor that plays a critical role in cognitive aging. Poor sleep quality has been linked to faster decline in processing speed and, in older adults over 65, to steeper declines in verbal memory [19]. Other everyday lifestyle factors, such as alcohol consumption and smoking, also appear to have an impact on cognition. For example, older adults who both smoked and drank heavily showed a 36% faster rate of cognitive decline, roughly equivalent to adding two extra years of aging over a 10-year period, compared with non-smoking moderate drinkers [20].

Yet, for individuals already experiencing SCCs, the evidence remains incomplete. It is still unclear whether lifestyle and psychosocial factors exert the same influence on cognitive performance in this more vulnerable group or which of these factors might serve as the most relevant targets for prevention and early intervention. Sleep disturbance and physical inactivity are of particular interest in this group, because both may plausibly contribute not only to measured performance but also to the discrepancy between perceived and objective cognitive function that defines it.

To explore this issue, we analyzed baseline data from the N-HANCE study, which followed middle-aged and older adults reporting SCCs. We examined twelve lifestyle, psychosocial and demographic predictors, including physical activity, education, alcohol consumption, perceived stress, mood, and sleep quality, against eleven objective attention and memory outcomes, applying false discovery rate correction across the full family of comparisons and adjusting surviving associations for age, sex and educational attainment. This allows us to ask not only which factors are associated with cognitive performance in individuals with SCCs, but which associations remain once educational attainment, as a proxy of reserve, and aging are accounted for.

## 2. Materials and Methods

This study is based on baseline data from the N-HANCE study, a prospective observational cohort designed to investigate the effect of dietary supplementation on the attentional performance, cognition, and mental well-being of middle-aged and older healthy adults with subjective cognitive function complaints [21]. Participants were recruited between March 2023 and June 2025 from the local community and all study procedures took place at the RoNeuro Institute for Neurological Research and Diagnostic, Cluj Napoca, Romania. A total of 276 individuals were enrolled. Eligible participants were men and women aged 50–75 years who reported SCCs but did not show evidence of clinically significant cognitive impairment (Montreal Cognitive Assessment score >25). Eligibility additionally required a body weight of at least 50 kg for men and 45 kg for women and a body mass index between 18.0 and 29.9 kg/m^2^; consequently, no participant in this sample met criteria for obesity. MoCA scores were obtained at screening to establish eligibility and were not retained in the analysis database. The full set of inclusion and exclusion criteria for the N-HANCE study is described in the published protocol (ISRCTN10514154; doi: https://doi.org/10.1186/ISRCTN10514154). Subjective cognitive complaints were ascertained at screening through a face-to-face semi-structured clinical interview conducted by a clinician with each participant. During this interview the clinician systematically explored perceived changes in everyday cognitive functioning across specific cognitive domains, including memory, executive function, attention, language and visuospatial abilities. No standardized questionnaire was administered, no numerical cutoff was applied, and participants were not selected according to the Subjective Cognitive Decline Initiative (SCD-I) research criteria [22]. Eligibility rested on the clinician’s judgment that the participant reported perceived cognitive change. No minimum duration or persistence of the complaint was required and no specific reference period was imposed. Because complaints were ascertained in this way rather than against formal research criteria, we use the broader term “subjective cognitive complaints” throughout in preference to “subjective cognitive decline”, the terminology recommended by the SCD-I for individuals reporting persistent perceived decline alongside normal objective performance.

Cognitive performance was assessed with the following tasks. The Test of Attentional Performance (TAP) was used, using three subtests: *alertness* (phasic alertness subtest): measuring reaction time in response to auditory warning signals; *working memory* (difficulty level 3): assessing accuracy in updating and maintaining information under increased cognitive load; and *divided attention*: measuring response accuracy and reaction times under dual-task conditions [23]. The Wechsler Adult Intelligence Scale—Fourth Edition (WAIS-IV) was also used, more exactly *Digit Span Forward (DSF)* and *Digit Span Backward (DSB)* in order to measure short-term memory capacity and working memory, respectively [24].

As psychosocial measures we used the Perceived Stress Scale (PSS) [25], Brief Mood Introspection Scale (BMIS) [26] and Sleep Quality Scale (SQS) [27]. Participants also provided information on physical activity (recorded on a four-level scale: sedentary; moderate, 1–2 days/week; increased, 5–6 days/week; daily workout). The four-level ordinal variable was used in all analyses. For descriptive presentation, the two highest levels were combined owing to the small number of participants reporting daily activity (*n* = 6), yielding low (sedentary), moderate (1–2 days/week) and high (≥5 days/week) categories. Participants also provided information on alcohol consumption (dichotomized as any current alcohol consumption vs. no alcohol consumption), tobacco use, BMI and self-reported medical history (such as hypertension, anxiety, depression). Educational attainment was coded using International Standard Classification of Education (ISCED) levels and collapsed for descriptive purposes into low (ISCED 0–2), medium (ISCED 3–5) and high (ISCED 6–8).

For the purpose of this paper, we analyzed data collected exclusively at Visit 1 (baseline) of the N-HANCE study. All participants were included in the analysis regardless of their subsequent randomization group in the parent trial and no unblinding of allocation was performed.

The N-HANCE study was conducted in accordance with the Declaration of Helsinki and approved by the Ethics Committee of the “Iuliu Hațieganu” University of Medicine and Pharmacy, Cluj-Napoca, Romania (8 Victor Babeș Street; ref: AVZ 294; approval date: 28 November 2022; Tel.: +40 264 597256; email: etica.cercetare@umfcluj.ro). Written informed consent was obtained from all participants prior to study enrollment.

Statistical analyses were conducted using SAS OnDemand for Academics (version 9.4.), with correction for multiple testing performed in Microsoft Excel (Microsoft 365, version 16.89.1) and data visualization performed using Python 3 with the matplotlib library (version 3.10.8). Cognitive outcomes were analyzed as normed T-values, which are scaled such that higher values indicate better performance. TAP T-values below the instrument floor of 20 were recoded to 19, following the convention of the parent trial. Raw error and omission counts were near-perfectly correlated with their corresponding T-values (|ρ| = 0.98–1.00) and were therefore not analyzed separately. Associations between continuous or ordinal predictors and cognitive performance were examined using Spearman rank-order correlation coefficients (ρ), selected a priori because several predictors were ordinal and the underlying performance distributions were markedly skewed. Differences in cognitive outcomes between binary categorical groups (alcohol and tobacco consumption, history of depression and history of anxiety) were evaluated using the Mann–Whitney U test, with effect sizes expressed as rank-biserial correlations. To account for multiple testing, the Benjamini–Hochberg false discovery rate procedure was applied in Microsoft Excel across the full family of 132 predictor–outcome comparisons [28]. Associations are described as surviving correction where q < 0.05. There were no missing data on any analyzed variable and all 132 comparisons were therefore based on the full sample of 276 participants. To assess independence from demographic confounding, each association was additionally examined in a linear regression model adjusted for age, sex and educational attainment. These three covariates were selected a priori because each is an established correlate of cognitive test performance and each was plausibly associated with the lifestyle and psychosocial exposures examined, creating potential for confounding. Regression coefficients were expressed in outcome standard deviation units. For continuous predictors both predictor and outcome were standardized, so coefficients represent standardized regression coefficients; for the binary predictor (history of depression) the predictor was coded 0/1 and not standardized, so the coefficient represents the adjusted between-group difference in outcome standard deviation units. Statistical significance is identical under either parameterization. A two-tailed *p*-value < 0.05 was considered nominally significant. Regression assumptions were examined for each of the five adjusted models in which the association remained nominally significant after adjustment. Residual normality was assessed using the Shapiro–Wilk test, homoscedasticity using White’s test, multicollinearity using variance inflation factors and influential observations using Cook’s distance. These models were additionally re-estimated using heteroscedasticity-consistent (HC3) standard errors as a sensitivity analysis. This cross-sectional analysis of baseline data was exploratory and was not prespecified in the parent trial protocol, which is reflected in the correction applied across the full family of comparisons.

## 3. Results

The analyses were based on baseline data from 276 participants who completed the lifestyle and cognitive assessments. Sociodemographic and clinical characteristics of the sample are presented in Table 1.

Of the 132 predictor–outcome associations examined, 41 were significant at the uncorrected threshold and 19 survived Benjamini–Hochberg correction (Table 2). Educational attainment accounted for eight of the surviving associations and age for three. Higher perceived stress was associated with poorer performance on Digit Span Backward (ρ = −0.21, q = 0.006) and with more working memory omissions (ρ = −0.16, q = 0.047). Both attenuated after adjustment for age, sex and education, and only the Digit Span Backward association remained nominally significant (β = −0.11, *p* = 0.046).

A more positive mood, as measured by the Brief Mood Introspection Scale, was associated with better performance on the Digit Span Backward task at the uncorrected threshold (ρ = 0.15, *p* = 0.012). This association did not survive correction for multiple comparisons (q = 0.062) and was further attenuated after adjustment (β = 0.09, *p* = 0.087). It is therefore reported as exploratory.

Poorer sleep quality was associated with more consistent alertness performance under warning conditions (ρ = 0.19, q = 0.016). Because higher standard deviation T-values on the TAP indicate less variable performance, this association runs counter to the expected direction. It did not remain significant after adjustment for age, sex and education (β = 0.11, *p* = 0.064) and should be regarded as provisional.

A visual summary of the direction and magnitude of significant associations across the attention and memory measures assessed is presented in Figure 1.

A history of depression was associated with poorer alertness performance. Participants reporting a history of depression showed slower reaction times on the alertness subtest without warning signal (rank-biserial r = −0.54, q = 0.007) and reduced phasic alertness (r = −0.49, q = 0.016). These associations were essentially unchanged after adjustment for age, sex and education. Expressed as fully standardized coefficients, which are more directly comparable with the other predictors examined, the adjusted effects were β = −0.190 and −0.205 respectively (both *p* < 0.001), indicating that they are not attributable to demographic confounding. These findings are based on 14 participants reporting a history of depression and should be interpreted with corresponding caution.

Participants reporting current alcohol consumption (*n* = 14) performed better than non-consumers on several measures, including Digit Span Backward (median 8 vs. 5) and phasic alertness. None of these differences survived correction for multiple comparisons (lowest q = 0.056). Alcohol consumers were on average six years younger than non-consumers (55.7 vs. 61.7 years). Given the small exposed group, the dichotomous exposure measure and this substantial age difference, these differences are most plausibly attributable to confounding and are not interpreted further.

Higher BMI was associated with poorer memory span (Digit Span Forward ρ = −0.18, q = 0.019), an association that remained significant after adjustment for age, sex and education (β = −0.16, *p* = 0.005). Associations with Digit Span Backward and with working memory omissions were significant at the uncorrected threshold only. Because BMI was restricted by design to the range 18.0–29.9 kg/m^2^, these associations describe variation within the normal-to-overweight range rather than effects of obesity.

Higher levels of physical activity were associated with fewer divided attention omissions (ρ = 0.18, q = 0.018) and faster alertness responses (ρ = 0.16, q = 0.047). After adjustment for age, sex and education, the association with divided attention omissions remained statistically significant, although marginally so (β = 0.12, 95% CI 0.01 to 0.23, *p* = 0.041), whereas the association with alertness was attenuated to non-significance (β = 0.10, 95% CI −0.01 to 0.21, *p* = 0.089). The divided attention result lies close to the conventional threshold and was sensitive to the handling of below-floor T-scores and should therefore be interpreted with caution.

Educational attainment emerged as the strongest correlate across the memory and attention measures assessed, accounting for eight of the nineteen associations surviving correction. Higher education was associated with better performance on Digit Span Backward (ρ = 0.44), working memory omissions (ρ = 0.32), Digit Span Forward (ρ = 0.31), divided attention omissions (ρ = 0.29), and alertness measures (ρ = 0.21 to 0.29), all q < 0.01. Age was also independently associated with cognitive performance: older participants showed poorer memory span (Digit Span Forward ρ = −0.26, q < 0.001; Digit Span Backward ρ = −0.20, q = 0.010) and less consistent alertness (ρ = −0.19, q = 0.016).

As shown in Table 3, associations for depression history and BMI were minimally attenuated after adjustment, whereas those involving sleep quality and positive mood did not persist and physical activity retained only a marginal association with divided attention omissions. Examination of regression assumptions in the five models retained after adjustment indicated no collinearity and no undue influence of individual observations: variance inflation factors ranged from 1.01 to 1.09 across the adjusted models and no observation exceeded a Cook’s distance of 0.065. Residuals departed from normality in three models (Shapiro–Wilk *p* = 0.020, *p* = 0.004 and *p* < 0.001) and there was evidence of heteroscedasticity in one model (White’s test, *p* = 0.026). Given the sample size (*n* = 276), these departures from residual normality are unlikely to materially affect inference. Under heteroscedasticity-consistent (HC3) standard errors, the associations involving history of depression (both *p* < 0.001) and BMI (*p* = 0.012) remained statistically significant, whereas those involving perceived stress (*p* = 0.062) and physical activity (*p* = 0.059) no longer reached the conventional threshold, consistent with their description as marginal.

## 4. Discussion

Subjective cognitive complaints are increasingly recognized as early markers of cognitive susceptibility in middle-aged and older persons. The current study aimed to offer a better understanding on how these everyday behaviors and experiences relate to cognitive performance in people who already report SCCs. Our analysis found several important associations among lifestyle and psychosocial characteristics and performance on measures of attention, working memory and memory span. These findings are broadly consistent with previous research, while also indicating that several commonly reported lifestyle associations are substantially attenuated once demographic factors are taken into account. It should be emphasized that subjective cognitive complaints, and cognitive vulnerability more broadly, are unlikely to reflect any single dimension. Cognitive, affective, functional and physical factors interact in shaping both measured performance and the perception of decline, as illustrated in work on the neuropsychological correlates of pre-frailty in neurocognitive disorders [29]. Although that work concerned patients with neurocognitive disorders rather than individuals with SCCs, its multidimensional framing is conceptually relevant here.

Earlier research showed that chronic stress can generate significant changes in brain function and structure, such as the hippocampus, amygdala and prefrontal cortex, and alterations in neurotransmitter metabolism, leading to elevated levels of cortisol and catecholamines [30,31]. All these joint factors impact specific cognitive functions, such as attention, working memory, or executive capacities [30].

According to our results, higher perceived stress was associated with poorer backward memory span and with more working memory omissions. These findings are consistent with several publications in the literature. Korten et al. identified a strong correlation between stress and reduced processing speed, reduced semantic fluency, worse direct and delayed recall, and inferior Digit Span Backward performance in 64-100-year-old adults [32]. The results of Franks et al. were even more unsettling, as greater perceived stress was associated with poorer performance across all cognitive domains [33]. Moreover, it was demonstrated that stress accelerates cognitive decline [34]. In the present analysis the association between perceived stress and Digit Span Backward survived correction for multiple comparisons and remained nominally significant after adjustment for age, sex and education, although the adjusted effect was modest.

Affective disorders like depression have been consistently associated with poorer cognitive performance in episodic memory, executive function, or processing speed [35]. A meta-analysis by Rock et al. demonstrated significant negative associations between depression severity and these specific domains, suggesting that cognitive deficits may represent a core feature of depression, not only a secondary effect of low mood [36]. Moreover, depression-related cognitive impairments can persist even after the affective symptoms fade away, highlighting possible neurobiological or psychosocial mechanisms that extend beyond transient mood changes [37,38]. Our results, which showed a lower alertness performance in subjects with a history of depression, are consistent with these results from the literature and reflect that a depression susceptibility may be related to reduced attentional efficiency even in people who lack clinically demonstrated cognitive impairment. Three further considerations temper the interpretation of this finding. First, the exposure was recorded as a binary history of depression, and information on age at onset, number or severity of episodes, recency of the last episode, current depressive symptoms, treatment or remission status was not collected; “history of depression” is therefore a broad and heterogeneous category. Second, because depression is itself associated with the propensity to report cognitive difficulties, it may relate not only to objective performance but also to the likelihood of reporting complaints in the first place. In a sample defined by the presence of such complaints, this raises the possibility that the association operates partly through the reporting of symptoms rather than solely through performance. Third, the exposed group comprised only 14 participants, so the estimate is imprecise and should not be over-interpreted despite its apparent robustness to adjustment.

On the other hand, participants with a more positive mood performed better on the Digit Span Backward task. This observation may support the idea that affective well-being may serve as a resilience factor in the early stages of cognitive impairment. Similar ideas are found in the literature as well; higher levels of positive affect were associated with improved cognitive flexibility, problem-solving, and working memory [39,40]. Positive emotions and a positive mentality in older persons were linked to the long-term maintenance of executive function and a slower rate of cognitive decline [41]. In our data, however, the association between positive mood and Digit Span Backward did not survive correction for multiple comparisons and was further attenuated after adjustment, so this interpretation should be regarded as tentative.

Our findings regarding sleep were limited and, in direction, unexpected: poorer self-reported sleep quality was associated with more rather than less consistent alertness performance and this association did not persist after adjustment for age, sex and education. We therefore do not interpret it substantively. Given the established importance of sleep in memory consolidation and reconsolidation and the reported relation between sleep disturbance and cerebral amyloid accumulation [42,43] the null and counter-directional findings here more likely reflect the limitations of a single self-report measure than an absence of association. Future work in this cohort should examine sleep using objective measures and longitudinal outcomes.

Participants reporting alcohol consumption performed better on several attention and memory measures, but we do not interpret this as evidence of a protective effect. The exposed group comprised only 14 participants, alcohol use was recorded dichotomously so that dose could not be examined, and consumers were on average six years younger than non-consumers. No alcohol association survived correction for multiple comparisons. The reported prevalence of any current alcohol use (5.1%) is also considerably lower than expected for a community sample in this age range, suggesting substantial under-reporting. While the wider literature describes a complex, dose-dependent relationship between alcohol intake and cognition, with light-to-moderate consumption sometimes associated with better performance and heavy consumption with structural brain changes and increased dementia risk [44,45,46,47], the present measure cannot speak to that question.

Higher body mass index was associated with poorer memory span in our sample, and this association persisted after adjustment for age, sex and education, suggesting that higher BMI may be implicated in measurable cognitive differences among people with subjective cognitive complaints. An important qualification is that eligibility for the N-HANCE study restricted BMI to 18.0–29.9 kg/m^2^, so no participant met the criteria for obesity. Our findings therefore describe graded variation within the normal-to-overweight range, which is a more restricted claim than that supported by earlier population-based research linking midlife obesity to executive dysfunction, poorer memory and elevated dementia risk [48,49]. That an association is detectable even across this narrow range is nonetheless noteworthy and range restriction would be expected to attenuate rather than inflate the observed effect.

Physical activity showed associations with alertness and divided attention that survived correction for multiple comparisons. After adjustment for age, sex and education, the association with divided attention omissions remained significant but marginal (*p* = 0.041), while the association with alertness was attenuated to non-significance. This pattern suggests that, in this cohort, the apparent relationship between physical activity and attentional performance is partly accounted for by demographic factors, with at most a modest independent contribution. This contrasts with the broader literature, in which consistent physical activity is reported to improve cognitive performance and reduce the risk of cognitive decline in older adults, particularly in attention and executive control [50,51], and in which meta-analyses of randomized trials indicate benefits of combined resistance and aerobic exercise [15]. Possible explanations include the coarse four-level self-report measure used here, the cross-sectional design and the relatively restricted range of activity in this sample.

However, the most consistent correlate of performance across the attention and memory measures assessed was education level. Higher education was significantly associated with faster alertness, fewer working memory and attention errors, and better memory span performance. Our results are consistent with the cognitive reserve framework described by Stern, in which higher educational attainment is associated with greater resistance to age-related cognitive decline through more efficient use of neural resources. Educational attainment is used here as a proxy of cognitive reserve rather than as a direct measure of it, and other proxies such as occupational complexity, cognitive leisure activity and premorbid intellectual ability were not available in this cohort [52,53]. Also, other large population-based studies have repeatedly shown that people with more education score higher on cognitive tests and have a lower risk of dementia [10,54]. The findings of our study emphasized the significance of education, which may influence early cognitive vulnerability and may even diminish some of the effects of lifestyle and psychosocial risk factors. Age showed a comparable pattern, being independently associated with poorer memory span and less consistent alertness. Taken together, education and age, a proxy of cognitive reserve and an index of aging respectively, accounted for the majority of associations that survived both correction for multiple comparisons and adjustment for confounding, while most of the modifiable lifestyle variables did not. This is an important qualification on the prospects for late-life lifestyle intervention in this population [10,55]. A further distinction should be drawn between the cross-sectional lifestyle associations reported here and the mechanisms through which cognitive reserve is thought to operate. Current lifestyle variables measured at a single timepoint are not direct measures of accumulated reserve and the relationship between modifiable lifestyle factors and reserve remains an active area of investigation [56]. Our findings should therefore be read as associations between concurrent behaviors and concurrent performance, not as evidence about reserve-building processes.

Several limitations should be acknowledged. First, this analysis is cross-sectional and based solely on baseline data, so no causal inference is possible. Second, a large number of associations was examined; although we applied Benjamini–Hochberg correction across the full family of 132 comparisons, associations not surviving correction should be regarded as exploratory. Third, several exposed subgroups were small (alcohol use *n* = 14, history of depression *n* = 14, history of anxiety *n* = 20), so the corresponding estimates are imprecise. Fourth, lifestyle factors, sleep and medical history were self-reported and are subject to recall and social desirability bias, the low reported prevalence of alcohol use may suggest under-reporting, and physical activity was captured with a coarse four-level measure that recorded neither intensity nor duration and contained no response option between 2 and 5 days per week. Fifth, eligibility restricted BMI to 18.0–29.9 kg/m^2^, limiting generalizability to individuals with obesity, and MoCA scores were not retained in the analysis database, so global cognitive status could not be characterized or adjusted for. Sixth, participants were recruited at a single center as volunteers for a supplementation trial and were predominantly female (72.1%) and highly educated, which limits generalizability; screening on an education-sensitive instrument may additionally have shaped the composition of the sample with respect to educational attainment, which was our strongest correlate. Findings should be interpreted cautiously and confirmed in longitudinal analyses. A further limitation concerns the scope of the cognitive assessment. The battery was restricted to measures of alertness, divided attention, working memory and memory span, and did not assess episodic memory, language, visuospatial function or executive function beyond working memory. The findings should therefore not be generalized to cognitive performance more broadly. In addition, subjective cognitive complaints were not characterized using a standardized instrument, limiting comparability with studies applying formal SCD research criteria.

## 5. Conclusions

In this cohort of middle-aged and older adults with subjective cognitive complaints, educational attainment and age showed the strongest and most consistent associations with performance on the attention and memory measures assessed. Among potentially modifiable factors, a history of depression and higher BMI were independently associated with poorer performance after adjustment for age, sex and education, and higher perceived stress showed a modest independent association with working memory. Physical activity retained a marginal independent association with divided attention, whereas associations involving mood and sleep quality did not persist after adjustment. These findings suggest that, in individuals already reporting cognitive complaints, educational attainment, used here as a proxy of cognitive reserve, showed stronger associations with measured performance than current lifestyle variables did. Depressive history and metabolic status emerge as candidate risk or resilience markers requiring longitudinal confirmation rather than as established modifiable targets, particularly given the small number of participants reporting a history of depression and the cross-sectional design. Because these analyses are cross-sectional and cannot establish causality, longitudinal follow-up of the N-HANCE cohort will be required to determine whether these factors predict future cognitive decline and to inform personalized early intervention strategies aimed at preserving cognitive health in aging populations.

## Figures and Tables

**Figure 1:**
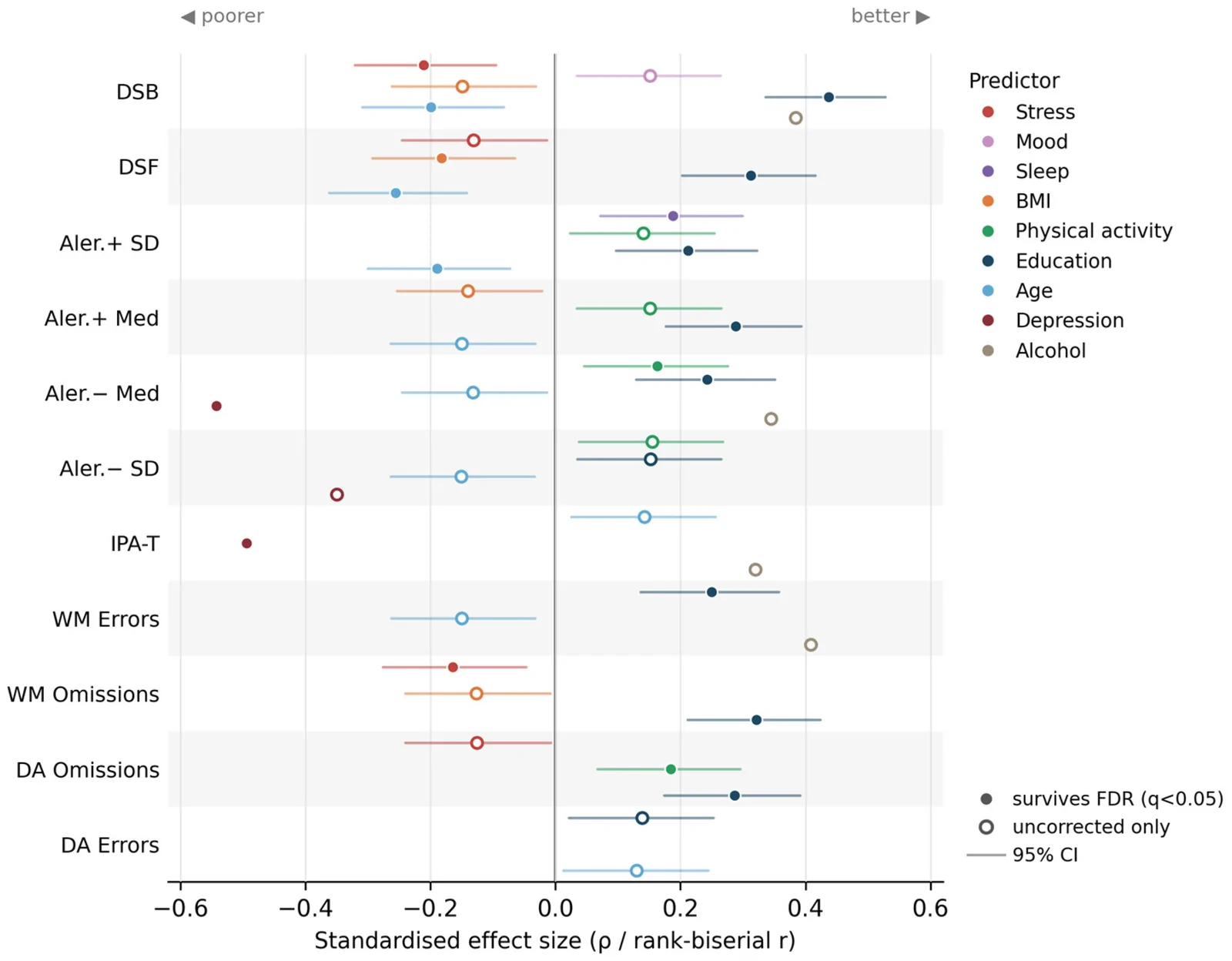
A coefficient plot of predictors of cognitive performance. The markers represent standardized effect sizes. For continuous and ordinal predictors (age, education, perceived stress, BMI, physical activity, sleep quality, mood), the values are Spearman rank-order correlation coefficients (ρ) shown with 95% confidence intervals. For binary clinical predictors (history of depression, alcohol consumption), the values are rank-biserial correlation coefficients. All cognitive outcomes are expressed as normed T-values scaled so that higher values indicate better performance; negative values therefore indicate associations with poorer cognitive performance and positive values associations with better performance. The filled markers denote associations surviving Benjamini–Hochberg correction across the full family of 132 predictor–outcome comparisons (q < 0.05); the open markers denote associations significant at the uncorrected threshold only (*p* < 0.05, q ≥ 0.05). The vertical solid line at 0 represents the null hypothesis of no association. Predictors with no association reaching the uncorrected threshold (tobacco use, sex and history of anxiety) are not displayed; the complete matrix of all 132 comparisons is provided in Supplementary Table S1. Abbreviations: Aler.− Med, alertness without warning signal (median T-value); Aler.− SD, alertness without warning signal (standard deviation T-value); Aler.+ Med, alertness with warning signal (median T-value); Aler.+ SD, alertness with warning signal (standard deviation T-value); DA Errors, divided attention errors; DA Omissions, divided attention omissions; DSB, Digit Span Backward; DSF, Digit Span Forward; IPA-T, index of phasic alertness (T-value); WM Errors, working memory errors; WM Omissions, working memory omissions. All outcomes are expressed as normed T-values scaled so that higher values indicate better performance.

**Table 1:** The baseline characteristics, psychosocial measures and cognitive performance of the study sample (N = 276).

Variable	Category	N (%) or Mean ± SD (Range)
Demographic and lifestyle characteristics		
Age (years)	–	61.4 ± 7.4 (50–75)
Sex	Female/Male	199 (72.1%)/77 (27.9%)
Education level	Low/Medium/High	39 (14.1%)/118 (42.8%)/119 (43.1%)
BMI (kg/m^2^)	–	27.0 ± 2.3 (20–29)
Physical activity level	Low/Moderate/High	87 (31.5%)/137 (49.6%)/52 (18.8%)
Sleep duration	<6 h/≥6 h	50 (18.1%)/226 (81.9%)
Tobacco use	Yes/No	45 (16.3%)/231 (83.7%)
Alcohol use	Yes/No	14 (5.1%)/262 (94.9%)
History of depression	Yes/No	14 (5.1%)/262 (94.9%)
History of anxiety	Yes/No	20 (7.2%)/256 (92.8%)
Psychosocial measures		
Perceived Stress Scale (PSS)	–	16.2 ± 6.5 (1–37)
Brief Mood Introspection Scale (BMIS)	–	12.4 ± 8.3 (-14–24)
Sleep Quality Scale (SQS)	–	15.0 ± 11.9 (0–55)
Attention (TAP, T-values)		
Alertness without warning signal	Median	37.0 ± 8.7 (19–68)
Alertness without warning signal	SD	43.5± 10.2 (19–77)
Alertness with warning signal	Median	38.1 ± 8.8 (20–72)
Alertness with warning signal	SD	41.7± 11.0 (19–71)
Index of phasic alertness	–	48.0 ± 12.0 (19–80)
Divided attention	Errors	47.0 ± 11.1 (19–70)
Divided attention	Omissions	41.5 ± 10.3 (19–64)
Working memory	Errors	50.3 ± 8.6 (24–57)
Working memory	Omissions	42.8 ± 14.0 (19–76)
Memory span (WAIS-IV, raw scores)		
Digit Span Forward	–	7.5 ± 2.1 (4–16)
Digit Span Backward	–	5.4 ± 1.8 (2–10)

Education was recorded using International Standard Classification of Education (ISCED) levels and collapsed into low (ISCED 0–2), medium (ISCED 3–5) and high (ISCED 6–8). Physical activity was recorded on a four-level scale and collapsed into low (sedentary), moderate (1–2 days/week) and high (≥5 days/week). PSS, Perceived Stress Scale (higher scores indicate greater perceived stress); BMIS, Brief Mood Introspection Scale (higher scores indicate more positive mood); SQS, Sleep Quality Scale (higher scores indicate poorer sleep quality); TAP, Test of Attentional Performance; SD, standard deviation of reaction time. TAP T-values are normed to a population mean of 50 and standard deviation of 10 and are scaled so that higher values indicate better performance. Digit Span values are raw total scores.

**Table 2:** Associations between predictors and cognitive performance surviving correction for multiple comparisons.

Predictor	Cognitive Outcome	Effect	95% CI	*p*	q
Age	Digit Span Forward	−0.26	−0.36 to −0.14	<0.001	<0.001
Age	Digit Span Backward	−0.20	−0.31 to −0.08	<0.001	0.010
Age	Alertness + Signal SD T	−0.19	−0.30 to −0.07	0.002	0.016
BMI	Digit Span Forward	−0.18	−0.29 to −0.07	0.002	0.019
Education (ISCED)	Digit Span Backward	+0.44	+0.34 to +0.53	<0.001	<0.001
Education (ISCED)	Working Mem. Omissions T	+0.32	+0.21 to +0.42	<0.001	<0.001
Education (ISCED)	Digit Span Forward	+0.31	+0.20 to +0.42	<0.001	<0.001
Education (ISCED)	Alertness + Signal Median T	+0.29	+0.18 to +0.39	<0.001	<0.001
Education (ISCED)	Divided Att. Omissions T	+0.29	+0.17 to +0.39	<0.001	<0.001
Education (ISCED)	Working Mem. Errors T	+0.25	+0.14 to +0.36	<0.001	<0.001
Education (ISCED)	Alertness -Signal Median T	+0.24	+0.13 to +0.35	<0.001	<0.001
Education (ISCED)	Alertness + Signal SD T	+0.21	+0.10 to +0.32	<0.001	0.006
History of depression	Alertness -Signal Median T	−0.54		<0.001	0.007
History of depression	Phasic Alertness Index T	−0.49		0.002	0.016
Perceived stress (PSS)	Digit Span Backward	−0.21	−0.32 to −0.09	<0.001	0.006
Perceived stress (PSS)	Working Mem. Omissions T	−0.16	−0.28 to −0.05	0.006	0.047
Physical activity	Divided Att. Omissions T	+0.18	+0.07 to +0.30	0.002	0.018
Physical activity	Alertness -Signal Median T	+0.16	+0.05 to +0.28	0.007	0.047
Sleep quality (SQS)	Alertness + Signal SD T	+0.19	+0.07 to +0.30	0.002	0.016

Spearman rank-order correlations (ρ) for continuous and ordinal predictors; rank-biserial correlations (r) for binary predictors. Cognitive outcomes are normed T-values scaled so that higher values indicate better performance. q-values derive from Benjamini–Hochberg correction across the full family of 132 predictor–outcome comparisons; all associations shown meet q < 0.05. CI, confidence interval (Fisher z transformation; not computed for rank-biserial correlations). The complete matrix of tested associations, including those not surviving correction, is provided in Supplementary Table S1.

**Table 3:** Associations before and after adjustment for age, sex and educational attainment.

Predictor	Cognitive Outcome	Unadjusted	Adjusted	95% CI	*p*	Attenuation	HC3 *p*
History of depression	Alertness − signal, median	−0.921	−0.866	−1.37 to −0.36	<0.001	6%	<0.001
History of depression	Index of phasic alertness	−0.925	−0.933	−1.46 to −0.41	<0.001	−1%	<0.001
BMI	Digit Span Forward	−0.252	−0.162	−0.28 to −0.05	0.005	36%	0.012
Perceived stress	Digit Span Backward	−0.199	−0.110	−0.22 to −0.00	0.046	44%	0.062
Perceived stress	Working memory, omissions	−0.131	−0.067	−0.18 to +0.05	0.269	49%	-
Physical activity	Divided attention, omissions	+0.167	+0.120	+0.01 to +0.23	0.041	28%	0.059
Physical activity	Alertness − signal, median	+0.146	+0.100	−0.01 to +0.21	0.089	31%	-
Sleep quality	Alertness + signal, SD	+0.133	+0.111	−0.01 to +0.23	0.064	17%	-
Positive mood	Digit Span Backward	+0.130	+0.092	−0.01 to +0.20	0.087	29%	-

The coefficients are expressed in outcome standard deviation units. For continuous predictors (BMI, perceived stress, physical activity, sleep quality, positive mood) both predictor and outcome were standardized, so the coefficients are standardized regression coefficients and express the change in outcome per one standard deviation of the predictor. For the binary predictor (history of depression, coded 0 = absent, 1 = present) the predictor was not standardized, so the coefficient expresses the adjusted difference between groups in outcome standard deviation units. Statistical significance is identical under either parameterization; the fully standardized equivalents for the depression rows are −0.190 and −0.205 respectively. Outcomes are coded so that higher values indicate better performance, and TAP T-scores below the instrument floor of 20 were recoded to 19 as in the parent trial. Attenuation expresses the proportional reduction in the absolute magnitude of the coefficient after adjustment. CI, confidence interval; HC3 *p*, *p*-value under heteroscedasticity-consistent (HC3) standard errors, computed for the models retained after adjustment; -, not re-estimated.

## Data Availability

The original study protocol is publicly available via the ISRCTN registry (ISRCTN10514154). De-identified participant data and analysis code will be made available by the corresponding author upon reasonable request.

## Supplementary Materials

The following supporting information can be downloaded at https://www.mdpi.com/article/10.3390/brainsci16101033/s1, Table S1: Complete matrix of tested associations between predictors and cognitive performance (N = 276; 132 comparisons).
