# Supplementary material for: Lifestyle, Psychosocial and Demographic Correlates of Objective Cognitive Performance in Middle-Aged and Older Adults with Subjective Cognitive Complaints: A Cross-Sectional Baseline Analysis of the N-HANCE Cohort
Source: Brain Sci. 2026 Sep 27;16(10):1033. doi: 10.3390/brainsci16101033 (PMC13702195; doi:10.3390/brainsci16101033)
Supplement: Supplementary file 1 [file brainsci-16-01033-s001.zip › brainsci-4554258-supplementary.pdf]

Supplementary Table S1. Complete matrix of tested associations between predictors and cognitive performance (N = 276; 132 comparisons).

All predictor–outcome comparisons examined in the study are reported, including those not reaching statistical significance. Effect sizes are Spearman rank-order correlation coefficients (ϱ) for continuous and ordinal predictors and rank-biserial correlation coefficients for binary predictors. Cognitive outcomes are normed T-values scaled so that higher values indicate better performance, except Digit Span, which is reported as raw total scores; positive effect sizes therefore indicate associations with better performance throughout.

Cognitive outcome	Effect size	95% CI	p	q (FDR)	Survives
Education					
Digit Span Backward	+0.437	+0.34 to +0.53	<0.001	<0.001	Yes
Digit Span Forward	+0.313	+0.20 to +0.42	<0.001	<0.001	Yes
Alertness −signal, median	+0.243	+0.13 to +0.35	<0.001	<0.001	Yes
Alertness −signal, SD	+0.152	+0.03 to +0.27	0.011	0.062	–
Alertness +signal, median	+0.288	+0.18 to +0.39	<0.001	<0.001	Yes
Alertness +signal, SD	+0.212	+0.10 to +0.32	<0.001	0.006	Yes
Index of phasic alertness	-0.063	-0.18 to +0.06	0.299	0.499	–
Working memory, errors	+0.250	+0.14 to +0.36	<0.001	<0.001	Yes
Working memory, omissions	+0.322	+0.21 to +0.42	<0.001	<0.001	Yes
Divided attention, omissions	+0.286	+0.17 to +0.39	<0.001	<0.001	Yes
Divided attention, errors	+0.139	+0.02 to +0.25	0.021	0.085	–
Age					
Digit Span Backward	-0.199	-0.31 to -0.08	<0.001	0.010	Yes
Digit Span Forward	-0.256	-0.36 to -0.14	<0.001	<0.001	Yes
Alertness −signal, median	-0.132	-0.25 to -0.01	0.029	0.106	–
Alertness −signal, SD	-0.151	-0.26 to -0.03	0.012	0.062	–
Alertness +signal, median	-0.150	-0.26 to -0.03	0.012	0.062	–
Alertness +signal, SD	-0.189	-0.30 to -0.07	0.002	0.016	Yes
Index of phasic alertness	+0.142	+0.02 to +0.26	0.018	0.079	–
Working memory, errors	-0.150	-0.26 to -0.03	0.013	0.062	–
Working memory, omissions	-0.039	-0.16 to +0.08	0.518	0.721	–
Divided attention, omissions	-0.061	-0.18 to +0.06	0.312	0.507	–
Divided attention, errors	+0.130	+0.01 to +0.24	0.031	0.107	–
Perceived stress (PSS)					
Digit Span Backward	-0.211	-0.32 to -0.09	<0.001	0.006	Yes
Digit Span Forward	-0.131	-0.25 to -0.01	0.029	0.106	–
Alertness −signal, median	-0.092	-0.21 to +0.03	0.126	0.283	–
Alertness −signal, SD	+0.008	-0.11 to +0.13	0.899	0.920	–
Alertness +signal, median	-0.073	-0.19 to +0.05	0.228	0.407	–
Alertness +signal, SD	+0.016	-0.10 to +0.13	0.797	0.863	–
Index of phasic alertness	+0.023	-0.09 to +0.14	0.698	0.796	–
Working memory, errors	-0.030	-0.15 to +0.09	0.622	0.767	–
Working memory, omissions	-0.164	-0.28 to -0.05	0.006	0.047	Yes
Divided attention, omissions	-0.126	-0.24 to -0.01	0.037	0.122	–
Divided attention, errors	+0.044	-0.07 to +0.16	0.470	0.681	–
Positive mood (BMIS)					
Digit Span Backward	+0.151	+0.03 to +0.26	0.012	0.062	–
Digit Span Forward	+0.083	-0.04 to +0.20	0.171	0.351	–
Alertness −signal, median	+0.074	-0.04 to +0.19	0.223	0.404	–
Alertness −signal, SD	-0.053	-0.17 to +0.07	0.377	0.585	–
Alertness +signal, median	+0.082	-0.04 to +0.20	0.175	0.351	–
Alertness +signal, SD	-0.076	-0.19 to +0.04	0.207	0.389	–
Index of phasic alertness	-0.038	-0.16 to +0.08	0.532	0.724	–
Working memory, errors	+0.034	-0.08 to +0.15	0.569	0.759	–

Working memory, omissions	+0.094	-0.02 to +0.21	0.120	0.273	–
Divided attention, omissions	+0.072	-0.05 to +0.19	0.231	0.407	–
Divided attention, errors	-0.114	-0.23 to +0.00	0.059	0.186	–
Sleep quality (SQS)					
Digit Span Backward	-0.082	-0.20 to +0.04	0.177	0.351	–
Digit Span Forward	+0.028	-0.09 to +0.15	0.640	0.775	–
Alertness −signal, median	-0.013	-0.13 to +0.10	0.825	0.871	–
Alertness −signal, SD	+0.103	-0.02 to +0.22	0.088	0.231	–
Alertness +signal, median	+0.081	-0.04 to +0.20	0.182	0.353	–
Alertness +signal, SD	+0.188	+0.07 to +0.30	0.002	0.016	Yes
Index of phasic alertness	-0.108	-0.22 to +0.01	0.074	0.208	–
Working memory, errors	+0.000	-0.12 to +0.12	0.999	0.999	–
Working memory, omissions	-0.048	-0.17 to +0.07	0.424	0.635	–
Divided attention, omissions	+0.008	-0.11 to +0.13	0.896	0.920	–
Divided attention, errors	+0.031	-0.09 to +0.15	0.609	0.760	–
BMI					
Digit Span Backward	-0.149	-0.26 to -0.03	0.013	0.063	–
Digit Span Forward	-0.182	-0.29 to -0.07	0.002	0.019	Yes
Alertness −signal, median	-0.098	-0.21 to +0.02	0.103	0.252	–
Alertness −signal, SD	-0.044	-0.16 to +0.07	0.467	0.681	–
Alertness +signal, median	-0.140	-0.25 to -0.02	0.020	0.083	–
Alertness +signal, SD	-0.065	-0.18 to +0.05	0.284	0.480	–
Index of phasic alertness	+0.101	-0.02 to +0.22	0.095	0.242	–
Working memory, errors	-0.031	-0.15 to +0.09	0.604	0.760	–
Working memory, omissions	-0.126	-0.24 to -0.01	0.036	0.122	–
Divided attention, omissions	-0.073	-0.19 to +0.04	0.224	0.404	–
Divided attention, errors	+0.006	-0.11 to +0.12	0.915	0.929	–
Physical activity					
Digit Span Backward	-0.002	-0.12 to +0.12	0.980	0.988	–
Digit Span Forward	+0.081	-0.04 to +0.20	0.178	0.351	–
Alertness −signal, median	+0.163	+0.05 to +0.28	0.007	0.047	Yes
Alertness −signal, SD	+0.155	+0.04 to +0.27	0.010	0.062	–
Alertness +signal, median	+0.151	+0.03 to +0.26	0.012	0.062	–
Alertness +signal, SD	+0.141	+0.02 to +0.25	0.019	0.082	–
Index of phasic alertness	+0.031	-0.09 to +0.15	0.610	0.760	–
Working memory, errors	+0.061	-0.06 to +0.18	0.315	0.507	–
Working memory, omissions	+0.096	-0.02 to +0.21	0.113	0.271	–
Divided attention, omissions	+0.184	+0.07 to +0.30	0.002	0.018	Yes
Divided attention, errors	+0.023	-0.10 to +0.14	0.709	0.796	–
History of depression (n exposed = 14)					
Digit Span Backward	-0.154	–	0.324	0.510	–
Digit Span Forward	-0.094	–	0.552	0.744	–
Alertness −signal, median	-0.543	–	<0.001	0.007	Yes
Alertness −signal, SD	-0.350	–	0.028	0.106	–
Alertness +signal, median	-0.297	–	0.062	0.189	–
Alertness +signal, SD	-0.126	–	0.428	0.635	–
Index of phasic alertness	-0.494	–	0.002	0.016	Yes
Working memory, errors	-0.256	–	0.099	0.246	–
Working memory, omissions	-0.216	–	0.173	0.351	–
Divided attention, omissions	-0.064	–	0.686	0.796	–
Divided attention, errors	+0.246	–	0.120	0.273	–
History of anxiety (n exposed = 20)					
Digit Span Backward	+0.032	–	0.811	0.865	–

Digit Span Forward	+0.233	–	0.080	0.219	–
Alertness −signal, median	-0.065	–	0.631	0.771	–
Alertness −signal, SD	+0.057	–	0.670	0.789	–
Alertness +signal, median	+0.074	–	0.581	0.759	–
Alertness +signal, SD	+0.149	–	0.269	0.462	–
Index of phasic alertness	-0.232	–	0.084	0.227	–
Working memory, errors	-0.130	–	0.321	0.510	–
Working memory, omissions	-0.026	–	0.849	0.882	–
Divided attention, omissions	+0.087	–	0.519	0.721	–
Divided attention, errors	+0.108	–	0.421	0.635	–
Alcohol use (n exposed = 14)					
Digit Span Backward	+0.384	–	0.014	0.064	–
Digit Span Forward	+0.264	–	0.093	0.241	–
Alertness −signal, median	+0.345	–	0.030	0.106	–
Alertness −signal, SD	+0.290	–	0.067	0.193	–
Alertness +signal, median	+0.175	–	0.270	0.462	–
Alertness +signal, SD	+0.100	–	0.531	0.724	–
Index of phasic alertness	+0.320	–	0.044	0.141	–
Working memory, errors	+0.409	–	0.008	0.056	–
Working memory, omissions	+0.047	–	0.767	0.837	–
Divided attention, omissions	+0.059	–	0.711	0.796	–
Divided attention, errors	+0.135	–	0.396	0.608	–
Tobacco use (n exposed = 45)					
Digit Span Backward	+0.022	–	0.813	0.865	–
Digit Span Forward	-0.041	–	0.664	0.789	–
Alertness −signal, median	-0.067	–	0.477	0.681	–
Alertness −signal, SD	+0.051	–	0.591	0.759	–
Alertness +signal, median	-0.067	–	0.480	0.681	–
Alertness +signal, SD	+0.096	–	0.310	0.507	–
Index of phasic alertness	-0.052	–	0.581	0.759	–
Working memory, errors	-0.049	–	0.592	0.759	–
Working memory, omissions	-0.122	–	0.194	0.372	–
Divided attention, omissions	-0.173	–	0.066	0.193	–
Divided attention, errors	-0.034	–	0.717	0.796	–
Sex (male vs female) (n exposed = 77)					
Digit Span Backward	+0.029	–	0.703	0.796	–
Digit Span Forward	-0.035	–	0.650	0.780	–
Alertness −signal, median	+0.143	–	0.066	0.193	–
Alertness −signal, SD	-0.107	–	0.166	0.351	–
Alertness +signal, median	+0.016	–	0.837	0.877	–
Alertness +signal, SD	-0.104	–	0.178	0.351	–
Index of phasic alertness	-0.030	–	0.699	0.796	–
Working memory, errors	+0.113	–	0.137	0.300	–
Working memory, omissions	+0.121	–	0.119	0.273	–
Divided attention, omissions	-0.095	–	0.221	0.404	–
Divided attention, errors	+0.024	–	0.754	0.829	–

q-values were obtained using the Benjamini–Hochberg false discovery rate procedure applied across the full family of 132 comparisons. "Survives" indicates q < 0.05. Nineteen associations survived correction. Confidence intervals for Spearman coefficients were derived using the Fisher z transformation and are not reported for rank-biserial coefficients. For binary predictors, n exposed denotes the number of participants in the exposed group; the comparison group comprises the remainder of the sample. CI, confidence interval; FDR, false discovery rate; SD, standard deviation of reaction time; −signal, without warning signal; +signal, with warning signal.
